# Cell Type-Specific Functions of *Period* Genes Revealed by Novel Adipocyte and Hepatocyte Circadian Clock Models

**DOI:** 10.1371/journal.pgen.1004244

**Published:** 2014-04-03

**Authors:** Chidambaram Ramanathan, Haiyan Xu, Sanjoy K. Khan, Yang Shen, Paula J. Gitis, David K. Welsh, John B. Hogenesch, Andrew C. Liu

**Affiliations:** 1 Department of Biological Sciences, University of Memphis, Memphis, Tennessee, United States of America; 2 Department of Psychiatry, University of California, San Diego, La Jolla, California, United States of America; 3 Center for Chronobiology, University of California, San Diego, La Jolla, California, United States of America; 4 Veterans Affairs San Diego Healthcare System, San Diego, California, United States of America; 5 Department of Pharmacology and Institute for Translational Medicine and Therapeutics, University of Pennsylvania School of Medicine, Philadelphia, Pennsylvania, United States of America; 6 Feinstone Genome Research Center, University of Memphis, Memphis, Tennessee, United States of America; RIKEN, Japan

## Abstract

In animals, circadian rhythms in physiology and behavior result from coherent rhythmic interactions between clocks in the brain and those throughout the body. Despite the many tissue specific clocks, most understanding of the molecular core clock mechanism comes from studies of the suprachiasmatic nuclei (SCN) of the hypothalamus and a few other cell types. Here we report establishment and genetic characterization of three cell-autonomous mouse clock models: 3T3 fibroblasts, 3T3-L1 adipocytes, and MMH-D3 hepatocytes. Each model is genetically tractable and has an integrated luciferase reporter that allows for longitudinal luminescence recording of rhythmic clock gene expression using an inexpensive off-the-shelf microplate reader. To test these cellular models, we generated a library of short hairpin RNAs (shRNAs) against a panel of known clock genes and evaluated their impact on circadian rhythms. Knockdown of *Bmal1*, *Clock*, *Cry1*, and *Cry2* each resulted in similar phenotypes in all three models, consistent with previous studies. However, we observed cell type-specific knockdown phenotypes for the *Period* and *Rev-Erb* families of clock genes. In particular, *Per1* and *Per2*, which have strong behavioral effects in knockout mice, appear to play different roles in regulating period length and amplitude in these peripheral systems. *Per3*, which has relatively modest behavioral effects in knockout mice, substantially affects period length in the three cellular models and in dissociated SCN neurons. In summary, this study establishes new cell-autonomous clock models that are of particular relevance to metabolism and suitable for screening for clock modifiers, and reveals previously under-appreciated cell type-specific functions of clock genes.

## Author Summary

Various aspects of our daily rhythms in physiology and behavior such as the sleep-wake cycle are regulated by endogenous circadian clocks that are present in nearly every cell. It is generally accepted that these oscillators share a similar biochemical negative feedback mechanism, consisting of transcriptional activators and repressors. In this study, we developed cell-autonomous, metabolically relevant clock models in mouse hepatocytes and adipocytes. Each clock model has an integrated luciferase reporter that allows for kinetic luminescence recording with an inexpensive microplate reader and thus is feasible for most laboratories. These models are amenable to high throughput screening of small molecules or genomic entities for impacts on cell-autonomous clocks relevant to metabolism. We validated these new models by RNA interference via lentivirus-mediated knockdown of known clock genes. As expected, we found that many core clock components have similar functions across cell types. To our surprise, however, we also uncovered previously under-appreciated cell type-specific functions of core clock genes, particularly *Per1*, *Per2*, and *Per3*. Because the circadian system is integrated with, and influenced by, the local physiology that is under its control, our studies provide important implications for future studies into cell type-specific mechanisms of various circadian systems.

## Introduction

In mammals, many aspects of daily behavior and physiology such as the sleep-wake cycle, body temperature, and liver metabolism are regulated by endogenous circadian clocks [1], [2]. The circadian time-keeping system is a hierarchical, multi-oscillator network, with the hypothalamic suprachiasmatic nucleus (SCN) acting as a central pacemaker at the top of the hierarchy. The SCN integrates external time cues and, through complex signaling cascades, synchronizes and coordinates extra-SCN oscillators in the brain and in peripheral clocks throughout the body, culminating in overt, coherent circadian rhythms at the organismal level [3], [4]. This time-keeping system is critical for normal physiology and behavior, and its disruption can lead to sleep disorders, metabolic syndrome, premature aging, and cancer (reviewed in [2], [5]).

Virtually all cells in our body have circadian oscillators [6]–[8]. Despite tissue-specific physiological differences, these oscillators share a highly conserved molecular mechanism – a negative feedback loop. This consists of transcriptional activators BMAL1 and CLOCK, which bind to E-box enhancers and activate the transcription of the *Per* and *Cry* families of repressors. These repressors then feed back to inhibit BMAL1/CLOCK activity and their own expression [9]. Each molecular component in the core clock loop is represented by multiple paralogs (*Bmal1, Bmal2; Clock, Npas2; Per1, Per2, Per3; Cry1, Cry2*), which provides the potential for functional redundancy and cell type specificity. In addition, post-translational modifications play critical roles in clock function. For example, the ubiquitin ligases FBXL3 and FBXL21 regulate period length and amplitude through ubiquitin-mediated degradation of CRY proteins and regulation of REV-ERBα activity [10]–[15].

This core clock loop integrates with other transcriptional systems such as the ROR/REV-ERB (via RORE) and DBP/E4BP4 (via D-box) accessory loops [16]. In the RORE loop, retinoic acid receptor-related orphan nuclear receptors (RORA, RORB, and RORC) act as activators, and REV-ERBs (REV-ERBα known as NR1D1 and REV-ERBβ known as NR1D2; referred to hereafter as NR1D1 and NR1D2) act as repressors to regulate rhythmic *Bmal1* expression via the RORE cis-element in the *Bmal1* promoter [17]–[19]. Similarly, DBP/TEF/HLF and E4BP4 serve as activators and repressors, respectively, to regulate D-box-mediated transcription of genes such as *Per3* [16], [19]. These interlocking loops mediated by E-box, RORE, and D-box cis-elements form a complex clock network. These loops act individually or in combination to give rise to distinct waves of gene transcription [16], [20]. For example, while *Nr1d1*, *Bmal1*, and *Per3* transcription are each mediated primarily by a single cis-element (i.e., primarily E-box, RORE, and D-box, respectively), many other clock genes (e.g., *Cry1*) are regulated via a combinatorial mechanism involving multiple circadian elements [21].

Cell-based models were instrumental in the identification and characterization of clock gene function in mammals [22], [23]. These studies relied on immortalized cell lines that display circadian rhythms of gene expression in a cell-autonomous manner (i.e., without systemic cues). We and others have used fibroblasts derived from clock component mutant mice expressing a clock gene reporter [17], [21], [24]. Cellular clock models for comprehensive genetic analysis, however, have so far been limited to 3T3 mouse fibroblasts and U2OS human osteosarcoma cells [22], [25], [26]. In the U2OS model, knockdowns of all clock components have been evaluated for impact on period length and amplitude [27]. In mice, 3T3 fibroblasts [22], [28] and more recently MMH-D3 hepatocytes [29] have been introduced as cellular clock models; however, unlike the U2OS model, these models haven't been fully characterized genetically.

An implicit assumption in all these studies is that the clock works the same way in all cell and tissue types, such that gene function determined in one cell or tissue type applies to all cells, regardless of local physiological inputs to the clock. However, while 3T3 cells may be an appropriate model of the fibroblast clock, it is likely not an appropriate model for other cells. Recent studies point to bidirectional interactions between circadian clocks and other cellular and physiological processes. Thus, the circadian system is integrated with, and influenced by, the local physiology. Of particular interest is the reciprocal interaction between clock function and metabolism [5], [30]–[33]. However, as yet, there aren't any characterized cellular models appropriate for the study of clock control of metabolism. Therefore, to reveal cell type-specific molecular, cellular, and physiological mechanisms of circadian clocks, new cell-autonomous, physiologically relevant peripheral clock models are needed.

To facilitate cell type-specific genetic characterization, we explored mouse cell lines relevant to major metabolic functions, focusing on 3T3-L1 adipocytes and MMH-D3 hepatocytes. The mouse 3T3-L1 adipocyte cell line reflects adipose tissue function and has been pivotal in advancing the understanding of basic cellular mechanisms associated with diabetes, obesity, and related disorders in thousands of studies (e.g., [34], [35]). In recent years, mouse MMH-D3 hepatocytes have become a prominent model reflecting hepatocyte function in the liver [36], [37]. Both cell lines have been shown to exhibit rhythmic expression of clock genes and other genes that are involved in and modulated by local physiology [29], [38], [39].

In this study, we used luciferase reporters of clock gene expression and established three high amplitude cell-autonomous clock models: 3T3 fibroblasts, 3T3-L1 adipocytes, and MMH-D3 hepatocytes, with 3T3 fibroblasts as a reference model. These reporter cells displayed persistent, high amplitude rhythms, which allowed longitudinal recording of clock gene rhythms with high temporal resolution using an inexpensive off-the-shelf microplate reader. For genetic perturbations, we developed a pipeline to produce high-quality lentiviral shRNAs to knock down any gene of interest, and validated these cellular models with shRNAs against a selected panel of known clock genes. We show that knockdown of many clock genes resulted in expected phenotypes in all tested cell lines. Unexpectedly, however, we also observed cell type-specific knockdown phenotypes, particularly within the *Per* gene family. This study has important implications for the tissue-specific mechanisms of circadian clocks.

## Results and Discussion

### Development of New Cell-Autonomous Clock Models

**Figure 1:**
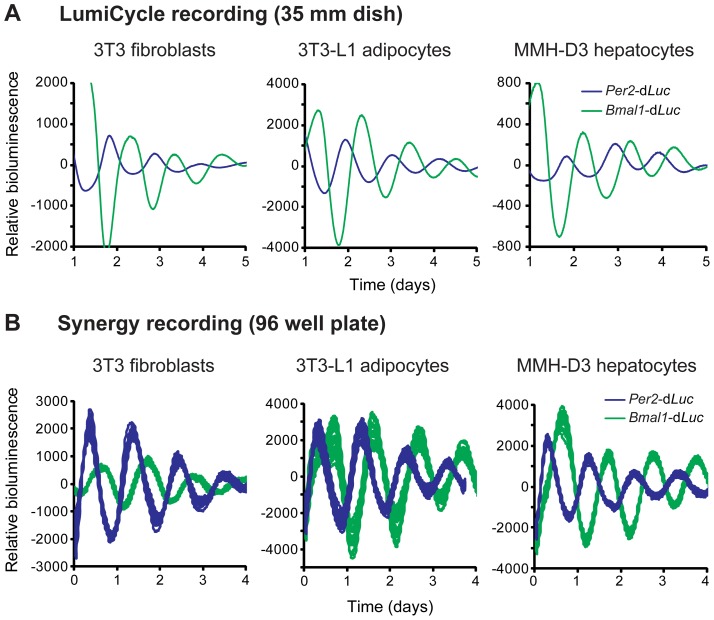
Fibroblasts, adipocytes, and hepatocytes display bioluminescence rhythms. (A) Representative bioluminescence rhythms of reporter cells recorded in a LumiCycle luminometer on 35 mm dishes. Reporter cells were generated via lentiviral infection of either *Per2*-d*Luc* or *Bmal1*-d*Luc* luciferase reporter, and infected cell populations were recorded in a LumiCycle. Baseline-subtracted bioluminescence data of both reporter lines are plotted together to show the expected, approximately anti-phasic reporter expression for each cell type. (B) Representative bioluminescence rhythms of homogenous clonal cell lines recorded in a Synergy microplate reader on 96 well plates. Baseline-subtracted bioluminescence data of selected clonal lines representing both reporter types are plotted together to show anti-phasic reporter expression for each cell type. High reproducibility is illustrated by showing overlapping traces from 24 of the 96 wells for each reporter. The period lengths are highly consistent (mean ± SD, n = 24 for each line): 3T3 *Per2*-d*Luc*, 25.62 hr±0.21; 3T3 *Bmal1*-d*Luc*, 26.72 hr±0.31; 3T3-L1 *Per2*-d*Luc*, 24.60 hr±0.32; 3T3-L1 *Bmal1*-d*Luc*, 25.01 hr±0.19; MMH-D3 *Per2*-d*Luc*, 24.49 hr±0.18; MMH-D3 *Bmal1*-d*Luc*, 25.33 hr±0.13.

As an initial effort to develop new cellular clock models pertinent to metabolism, we screened cell lines for robust rhythms and chose 3T3-L1 adipocytes and MMH-D3 hepatocytes. We introduced a lentiviral reporter harboring the rapidly degradable firefly luciferase (d*Luc*) gene under the control of either mouse *Per2* or *Bmal1* gene promoters into cells [23]. Whereas the 3T3 reporter cells were directly used in bioluminescence recording, 3T3-L1 and MMH-D3 cells were first differentiated into mature adipocytes and hepatocytes, respectively, prior to recording. These cells displayed persistent bioluminescence rhythms in 35 mm culture dishes monitored in a LumiCycle luminometer (Figure 1A). In each cell line, *Per2*-d*Luc* and *Bmal1*-d*Luc* reporters displayed anti-phasic rhythms of bioluminescence, consistent with the function of E-box- and RORE-containing promoters in regulating distinct and opposite phases of gene expression.

Next, we adapted the LumiCycle reporter assay to high-throughput screening (HTS) formats on 96 well plates. For this, we performed single cell cloning and selected clonal cell lines that expressed high levels of bioluminescence. These reporter lines displayed persistent rhythms under optimized growth conditions when monitored on a microplate reader (Synergy 2 SL) with highly consistent period lengths (Figure 1B). These highly reproducible rhythms seen in 96 well plates were similar to those in the LumiCycle, a lower throughput but much more expensive recorder. Therefore, these lines represent a tangible advantage to many labs interested in exploring circadian biology in these metabolically relevant cell lines.

### Generation of Lentiviral shRNAs for Gene Knockdown

For genetic perturbations, we developed a pipeline to produce high-quality, validated lentiviral shRNA vectors to knock down any mouse gene. We chose lentiviral shRNAs over transfected siRNAs because lentivirus-mediated delivery mediates potent transduction and stable integration in both dividing and non-dividing cells of various types *in vitro* and *in vivo*, thus circumventing low transfection efficiency for certain cells. We designed 6 target oligonucleotide sequences for each gene and cloned the shRNA expression cassette into the lentiviral pLL3.7GW Gateway vector, in which shRNA expression is under the control of the mouse U6 promoter, as we reported previously (Figure S1) [17]. Infectious lentiviral particles were produced in 293T cells using standard procedures and used to infect reporter cells. Infection efficiency was estimated by observing GFP co-expressed from a separate expression cassette under control of the CMV promoter (Figure S1).

We used this pipeline to generate a panel of shRNA constructs targeting the following selected 13 clock genes: *Bmal1*, *Bmal2*, *Clock*, *Npas2* (core loop activators); *Per1*, *Per2*, *Per3*, *Cry1*, *Cry2* (core loop repressors); *Fbxl3* (core loop post-translational modifier); *Nr1d1*, *Nr1d2* (RORE repressors); and *E4bp4* (D-box repressor). Because of the more prominent roles of repressors in clock function, we chose to examine *Nr1d1*, *Nr1d2*, and *E4bp4*, the RORE and D-box negative factors, rather than the corresponding activators [17], [40], [41].

We tested shRNA knockdown (KD) efficiency for the 13 clock genes. Co-transfection of shRNA with Flag-tagged cDNA in 293T cells followed by Western blot analysis showed efficient KD of each gene at the protein level by at least two shRNAs (Figure S2). To check the KD efficiency for endogenous gene expression, mRNA levels of targeted clock genes were also measured using qPCR. For each gene, at least two shRNAs were effective in knocking down gene expression, a requirement to filter out off-target effects of shRNAs [42]. The versatility and efficiency of lentiviral shRNA allowed us to study all 13 known clock genes in all three cell type-specific clock models in parallel, which allows direct phenotypic comparison.

### Ubiquitous Functions of *Bmal1*, *Clock*, *Cry1*, *Cry2*, and *Fbxl3*

**Figure 2:**
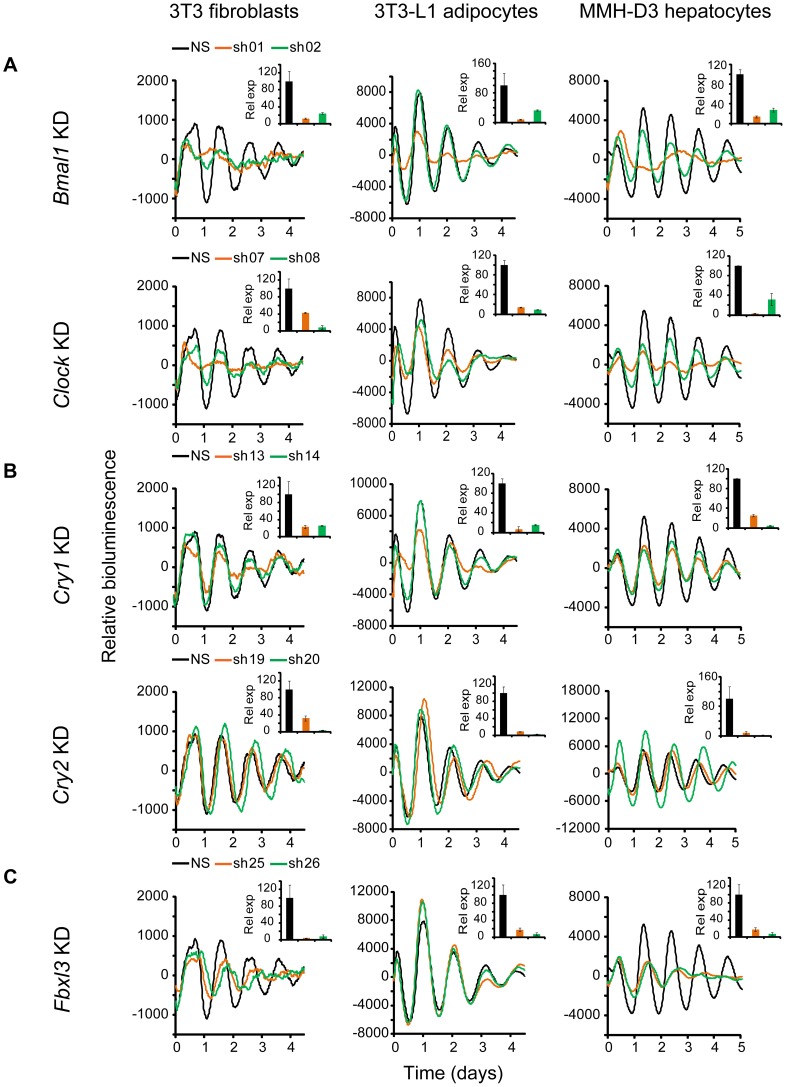
Knockdowns of *Bmal1*, *Clock*, *Cry1*, *Cry2*, and *Fbxl3* lead to cell type-ubiquitous circadian phenotypes. Bioluminescence expression patterns upon KD of *Bmal1* or *Clock* (A), *Cry1* or *Cry2* (B), and *Fbxl3* (C) in all three cell types. For clock phenotyping, both reporters were used for each cell line and phenotypes were independent of the reporter used. For phenotyping, we selected 3T3 cells expressing the *Bmal1*-d*Luc* reporter, and 3T3-L1 and MMH-D3 cells expressing the *Per2*-d*Luc* reporter. Cells were infected with specific lentiviral shRNAs as indicated. Real-time bioluminescence expression was recorded by Synergy microplate reader as in Figure 1. Out of the 6 shRNAs tested, two validated shRNAs (orange and green) are shown. NS, non-specific shRNA as control (black). While KD of *Bmal1* or *Clock* resulted in low amplitude, rapid damping or arrhythmicity, *Fbxl3* KD led to long period and low amplitude in 3T3, long period in 3T3-L1, and low amplitude and rapid damping in MMH-D3 cells. *Cry1* KD caused rapid damping or low amplitude, and *Cry2* KD lengthened period and increased rhythm amplitude. Bioluminescence data are representative of four independent experiments for 3T3 and 3T3-L1 cells, and three independent experiments for MMH-D3 cells. Knockdown of endogenous mRNA expression in non-synchronized cells was determined by qPCR (insert). Values for each gene are expressed as percentage of gene expression in NS control cells. qPCR data are mean ± SD (two samples/wells from one experiment).

Knockdown of *Bmal1*, *Clock*, *Cry1*, *Cry2*, and *Fbxl3* in all three cellular models resulted in expected phenotypes similar to those in LumiCycle assays using 35 mm dishes and consistent with previous knockout and knockdown studies using human and mouse cellular models [17], [27], [31], [43]–[45]. Specifically, KD of *Bmal1* or *Clock* results in rapid damping or arrhythmicity (Figure 2A and Tables 1, S1, S2, S3); *Cry1* KD leads to low amplitude or rapid damping depending on KD efficiency, whereas *Cry2* KD lengthens period and increases rhythm amplitude (Figure 2B). The phenotypic defects correlate with KD efficiency of the endogenous genes by the individual shRNAs as determined by qPCR analysis. Taken together, our data demonstrate that *Bmal1*, *Clock*, *Cry1*, and *Cry2* play similar roles in the clock mechanism across tested cell types, which provides validation for the three cellular models.

**Table 1:** Summary of knockdown phenotypes.

Gene KD	3T3 Fibroblasts	3T3-L1 Adipocytes	MMH-D3 Hepatocytes	U20S Osteosarcoma cells
***Bmal1***	AR	RD	AR	AR
***Bmal2***	WT	WT	WT	WT
***Clock***	AR	RD	AR	AR, LA
***Npas2***	WT	WT	WT	Short
***Cry1***	RD	RD	LA	Short
***Cry2***	Long	Long	Long, HA	Long
***Per1***	WT	WT	Short, LA	AR, LA
***Per2***	Short	WT	Short, LA	Long, LA
***Per3***	Short	Short	Short	Short
***Fbxl3***	Long, LA	Long	RD, LA	Long
***Nr1d1***	WT	WT	WT	Long
***Nr1d2***	LA	Short	Short	WT
***E4bp4***	Short, RD	LA	Short	Short

Notes:

1) Knockdown phenotypes in 3T3 fibroblasts, 3T3-L1 adipocytes, and MMH-D3 hepatocytes are from this study, and those in U2OS osteosarcoma cells are from [27], [31].

2) Detailed circadian parameter analyses and phenotypes in 3T3, 3T3-L1 and MMH-D3 cells are presented in Tables S1, S2, S3.

3) Phenotypes: WT, wild type; short, shorter period length than WT; long, longer period length than WT; LA, rhythmic but low amplitude; HA, rhythmic but high amplitude; RD, rapid damping (i.e., rapid decline in amplitude over time) and only transiently rhythmic; AR, arrhythmic.

Knockdown of *Bmal2* and *Npas2* did not exhibit any obvious circadian phenotypes in any of the three cellular models (Figure S3), even though their expression was knocked down to levels similar to those for *Bmal1* or *Clock* (Figure 2A and Table 1). These results are consistent with absence of observable circadian phenotypes of liver and lung tissues from *Npas2^−/−^* mice [46], though these mice do have deficits in circadian behavior and sleep homeostasis [47]. Despite potential functional redundancy between *Bmal1* and *Bmal2* [45], [48], and between *Clock* and *Npas2* [46], our data suggest that *Bmal2* and *Npas2* are not necessary for the clock to operate in these cells.

We show that *Fbxl3* KD caused long period and low amplitude in 3T3, long period in 3T3-L1, and low amplitude and rapid damping in MMH-D3 cells (Figure 2C). This cell-autonomous phenotype is much more extreme compared to the relatively modest period-lengthening phenotypes seen at the SCN tissue and behavioral levels [10]–[12], or in human U2OS cells [27], [44]. Notably, although KD of *Fbxl3* or *Cry2* both produced long periods, *Cry2* down-regulation increased rhythm amplitude, whereas *Fbxl3* silencing resulted in low amplitude, consistent with its dual role in ubiquitin-mediated degradation of CRY proteins and in regulation of NR1D1-mediated transcriptional suppression [10]–[13].

### *Nr1d1*, *Nr1d2*, and *E4bp4* Knockdown Caused Cell Type-Dependent Clock Phenotypes

**Figure 3:**
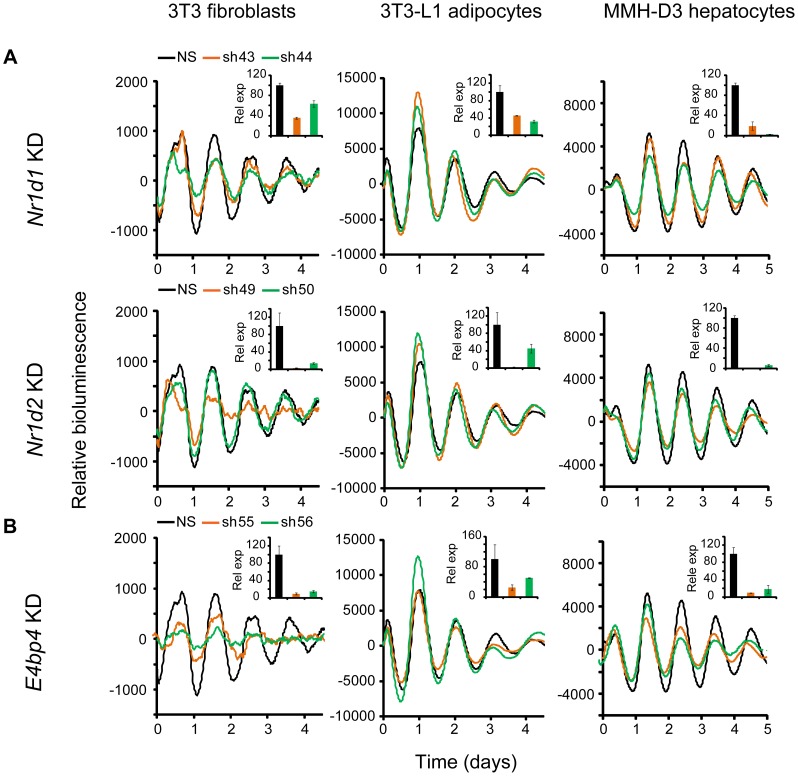
Knockdowns of *Nr1d1*, *Nr1d2*, and *E4bp4* lead to cell type-specific circadian phenotypes. Bioluminescence expression patterns upon KD of *Nr1d1* and *Nr1d2* (A) and *E4bp4* (B) in all three cell types. See Figure 2 for details. Note that, compared to the more prominent role of *Nr1d1* in clock function previously found in U2OS cells or mouse behavioral rhythms [18], [27], [49], *Nr1d2* plays a more prominent role in all three cells. *E4bp4* KD caused period length and/or amplitude phenotypes depending cell type.

*Nr1d1* and *Nr1d2* play overlapping but essential functions in regulating RORE-mediated transcription, and knockdown of either gene results in low amplitude rhythms, and in some cases, short period [17], [27]. We examined the effects of *Nr1d1* or *Nr1d2* KD on clock function in our cellular clock models. Knockdown of *Nr1d1* resulted in largely normal rhythms in these cells (Figure 3A and Table 1), indicating potential overlapping functions of *Nr1d2* [17]. This is different from the period lengthening produced by siRNA knockdown in U2OS cells [27], [31] or the period shortening and greater variability seen in behavioral rhythms of *Nr1d1^−/−^* mice [18], [49]. *Nr1d2* KD, on the other hand, resulted in period shortening in MMH-D3 and 3T3-L1 cells but low amplitude in 3T3 cells (Figure 3A and Table 1). The *Nr1d2* impact on overall rhythms was at least similar to, if not substantially stronger than, for *Nr1d1*. This is consistent with the reported redundant functions of *Nr1d1* and *Nr1d2* [17], but contrasts with previous studies showing that *NR1D2* deficiency has no observable rhythm phenotype in U2OS cells [27] or in mice at the behavioral level [18]. Thus, *Nr1d1* and *Nr1d2* play different roles in clock function depending on tissue or cell type, and *Nr1d2* may be more important than previously recognized.

Despite the widely accepted role of E4BP4 as the repressor of D-box-mediated transcription, definitive genetic evidence of clock function has been lacking. We show here that *E4bp4* KD resulted in short period and rapid damping in 3T3 fibroblasts, low amplitude in 3T3-L1 cells, and short period in MMH-D3 cells (Figure 3B and Table 1). These data are in line with recent studies suggesting a prominent role of *E4bp4* in regulating the phase of *Cry1* transcription in mouse embryonic fibroblasts [21] and period length in Rat-1 fibroblasts [41]. Studies of E4BP4's function in the clock mechanism using *E4bp4^−/−^* mice are therefore needed to validate our findings in cellular clock models.

### Cell Type-Specific Clock Functions of *Per1*, *Per2*, and *Per3*

**Figure 4:**
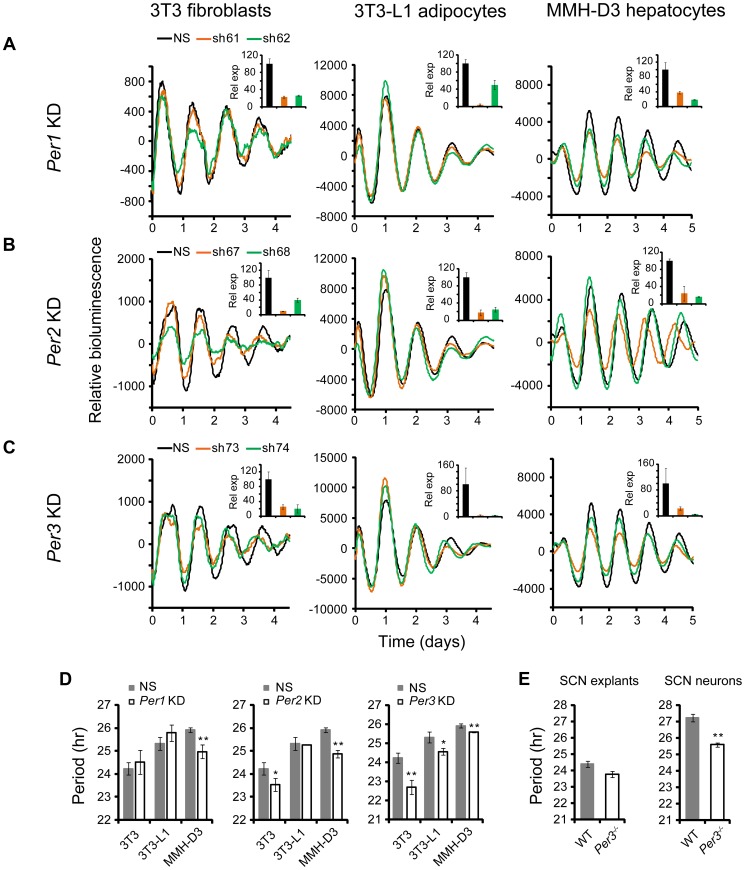
shRNA-mediated knockdowns of *Per1*, *Per2* and *Per3* lead to cell type-specific circadian phenotypes. Bioluminescence expression patterns upon KD of *Per1* (A), *Per2* (B), and *Per3* (C) in all three cell types. See Figure 2 for details. Whereas *Per3* KD led to short periods in all three cell types, *Per1* and *Per2* KDs caused different clock phenotypes depending on cell type. (D) Summary of period length phenotypes. Data are mean ± SD (n = 4 independent experiments for 3T3 cells; n = 3 samples/wells of one experiment for 3T3-L1 and MMH-D3 cells). NS, non-specific shRNA. Compared to NS controls, significant difference in period length was detected in the following KDs: NS vs. *Per1* KD in MMH-D3, t-test, p<0.001; NS vs. *Per2* KD in 3T3, t-test, p = 0.013; NS vs. *Per2* KD in MMH-D3, t-test, p<0.001; NS vs. *Per3* KD in 3T3, t-test, p<0.001; NS vs. *Per3* KD in 3T3-L1, t-test, p = 0.003; NS vs. *Per3* KD in MMH-D3, t-test, p<0.001). *p<0.01; ** p<0.001. (E) *Per3* deletion led to short period length defects in SCN explants (left) and even stronger defects in dissociated SCN neurons (right). *Per3^−/−^* SCN explants show a slightly shorter period than WT (mean ± SEM: WT, 24.4 hr±0.17, n = 5; *Per3^−/−^*, 23.78 hr±0.18, n = 5). The mean period of rhythms in *Per3^−/−^* neurons was substantially shorter than in WT cells (mean ± SEM: WT, 27.23 hr±0.24, n = 106; *Per3^−/−^*, 25.58 hr±0.12, n = 157; t-test, p<10E-10; ** p<0.001).

The shRNA constructs against *Per1*, *Per2*, and *Per3* down-regulated mRNA and protein expression (Figures S2 and 4). However, unlike *Cry1* and *Cry2* KDs, knockdown of the *Per* genes in our clock models resulted in cell type-specific clock phenotypes (Figure 4). First, compared to the dramatic circadian defects observed in peripheral tissue explants and fibroblasts of *Per1^−/−^* mice, or upon siRNA-mediated *Per1* KD in U2OS cells [31], [43], [50], *Per1* KD in our clock models had milder effects on clock function. Interestingly, these less dramatic phenotypes are cell type-specific: WT phenotype in 3T3 and 3T3-L1 cells, but significantly shorter period and low amplitude in MMH-D3 cells (Figures 4A and 4D; Table 1).

Similarly, *Per2* KD did not cause arrhythmicity in any of the three cellular clock models: only a modest reduction of amplitude in 3T3-L1 cells, but significantly shorter period in 3T3 and short period and low amplitude in MMH-D3 cells (Figures 4B and 4D; Table 1). The amplitude reduction in MMH-D3 cells was evident in both the subtracted data and raw data (compare Figure 4 with Figure S5). Although it is possible that the more modest phenotypes may be due to incomplete silencing, the knockdown levels were comparable to those of *Bmal1*, *Clock*, and *Fbxl3* (Figures 2 and 4B); and as in the case of *Per1* KD, the different phenotypes resulted from similar *Per2* KD efficiency in different cells. This is unexpected given its essential role in circadian rhythms of mice at the behavioral level and in cultured fibroblasts and U2OS cells [27], [43], [51]–[53]. Interestingly, however, our finding of non-essential but cell type-specific role of *Per2* is in line with a recent report showing that *Per2^−/−^* SCN explants displayed persistent rhythms with short periods, whereas *Per2^−/−^* pituitary explant rhythms were normal and lung explants displayed slightly long periods [54]. Thus, even though genetic knockout and knockdown (incomplete silencing) may cause variations in phenotypes, the loss-of-function phenotypes of *Per1* and *Per2* are largely consistent and are cell or tissue type specific.

While *Per2* appears to be more important than *Per1* for normal clock function in mice [52], [55], deletion of *Per3* has only subtle effects on the SCN clock and is often not considered part of the core clock mechanism [43], [54], [56]. However, we show here that KD of *Per3* in all three models produced significantly shorter periods than in control cells (Figures 4C and 4D; Table 1). These results are consistent with recent reports showing that tissue explants of *Per3^−/−^* mice, including liver, lung, and pituitary, also displayed short periods [43], [54]. In addition, knockdowns of *Per1*, *Per2*, and *Per3* showed similar phenotypes in cells expressing a different reporter (Figures 4 and S4), confirming that the knockdown effects are reporter independent. Furthermore, the *Per1* and *Per3* knockdown effects are largely consistent with data of liver explants from *Per1* and *Per3* knockouts [43], [57], [58].

### *Per3* Plays an Important Role in the SCN Clock

The prominent role of *Per3* in peripheral clock function led us to examine its function more carefully in both intact SCN explants and dissociated SCN neurons derived from *Per3^−/−^*:*mPer2^Luc^* mice. We detected persistent *mPer2^Luc^* rhythms in *Per3^−/−^* SCN explants with a slightly shorter period than WT (Figure 4E). This is consistent with the original study of *Per3^−/−^* mice, where a slightly shorter period of behavioral rhythms was reported [56]. We then dissociated *Per3^−/−^* SCN neurons and examined *mPer2^Luc^* bioluminescence from dispersed neurons at the single-cell level, as we have done previously for other genotypes [43]. We found that dissociated WT and *Per3^−/−^* neurons generally exhibited persistent rhythms with high amplitude. However, the mean period of rhythms in *Per3^−/−^* neurons was substantially shorter than in WT cells (Figure 4E). The weaker circadian defect at the SCN tissue level than in cell-autonomous preparations (either our cellular models or dissociated SCN neurons) is consistent with the principle that the SCN network confers robustness against genetic perturbations [43], [59], such that *Per3* plays a less prominent role in the intact SCN due to compensation by the SCN network. Based on these results, we conclude that *Per3* plays an important role in the SCN cellular clock as well as in peripheral oscillators and thus represents a *bona fide* clock component.

The more prominent role of *Per3* in peripheral oscillators is in line with several recent studies showing that disruption of *Per3* resulted in internal phase misalignment or desynchrony, or aberrant metabolic and sleep phenotypes [58], [60]–[62], all pointing to the role of *Per3* in coherence of circadian organization. Thus, our findings suggest that tissue-specific function or dysfunction of clock genes in peripheral tissues can be an important contributing factor to human diseases, even when the behavioral effect of gene knockout is subtle. In this context, it is interesting to note that human polymorphisms in *PER3* are associated with sleep and metabolic disorders [63], [64].

Taken together, our study expands our knowledge of the distinct functions of known clock genes across tissues. In particular, *Per3* plays a more important role in both SCN and non-SCN cells than previously appreciated, and *Per1* and *Per2* appear to have different roles in different cell types. Results from this study are broadly consistent with previous findings from loss-of-function studies and collectively point to the previously under-appreciated cell type specificity of *Per* gene function in circadian physiology (Figure S6).

### Composite Knockdown of the *Per* Genes in MMH-D3 Hepatocytes

**Figure 5:**
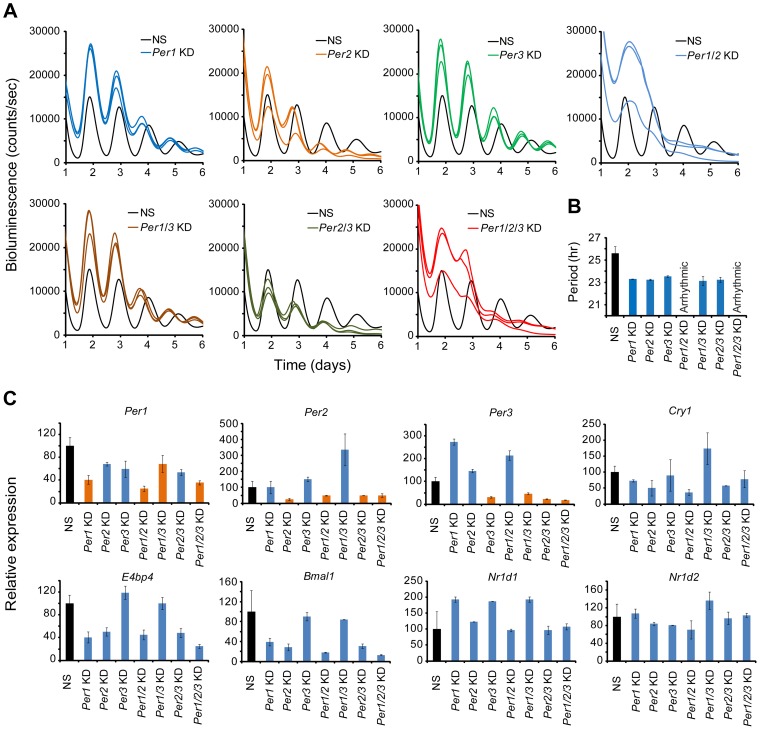
shRNA-mediated single and composite knockdown effects of *Per1*, *Per2* and *Per3* in MMH-D3 hepatocytes. (A) Representative bioluminescence expression patterns recorded on LumiCycle upon knockdowns of *Per1*, *Per2*, *Per3* (single KD); *Per1*/*Per2*, *Per1*/*Per3*, *Per2*/*Per3* (double KD); and *Per1*/*Per2*/*Per3* (triple KD) in MMH-D3 hepatocytes. sh62, sh67 and sh74 were used to knock down *Per1*, *Per2* and *Per3*, respectively. All single KDs led to short periods in all three cell types, consistent with Synergy assays. *Per1*/*Per2* double and *Per1*/*Per2*/*Per3* triple KDs caused arrhythmicity. All other double composite KDs caused short period phenotype. (B) Summary of period length phenotypes. Data are mean ± SD (n = 3 independent samples/wells from one experiment). NS, non-specific shRNA. Compared to NS controls, significant difference in period length was detected (NS vs. *Per1* KD, t-test, p<0.001; NS vs. *Per2* KD, t-test, p<0.001; NS vs. *Per3* KD, t-test, p<0.001; NS vs. *Per1*/*Per3* KD, t-test, p<0.001; NS vs. *Per2*/*Per3* KD, t-test, p<0.001). (C) qPCR analysis of clock gene expression upon *Per* KD in non-synchronized MMH-D3 cells. Values for each gene are expressed as percentage of gene expression in NS control cells. Data represent two samples/wells from one experiment.

Compared to other cell-autonomous models, the short period length in MMH-D3 cells after knockdown of each of the *Per* genes is unique (Figure S6 and Table 1), and therefore we sought to perform single and composite knockdowns for further phenotyping using the LumiCycle assay. Consistent with the Synergy assay, *Per1*, *Per2*, and *Per3* single gene KD each gave short period phenotypes, about 2 hrs shorter than the control cells (Figures 5A and 5B; Table S4). Composite *Per1*/*Per2* double KD and *Per1*/Per2/*Per3* triple KD caused complete arrhythmicity (Figure 5A), indicating the prominent roles of *Per1* and *Per2* in the hepatocyte clock. Interestingly, *Per1*/*Per3* and *Per2*/*Per3* double KDs did not cause any further period shortening over single *Per* gene KDs (Figures 5A and 5B).

As an initial effort to probe the network features of the hepatocyte clock, we examined effects of *Per* KD on the expression of endogenous clock genes by qPCR. *Per1*, *Per2*, and *Per3* were each knocked down in both single and composite KDs (Figure 5C). Compared to the U2OS model, paralog compensation among the *Per* genes in MMH-D3 cells is more pervasive. In our MMH-D3 model, both *Per1* KD and *Per2* KD upregulated *Per3*, and *Per3* KD upregulated *Per2*. While composite *Per1*/*Per2* KD upregulated *Per3* and *Per1*/*Per3* KD greatly upregulated *Per2*, *Per2*/*Per3* KD did not increase *Per1* expression. Interestingly, *Per1* and/or *Per2* KD had milder effects on the expression of E-box-containing genes (e.g., *Nr1d1* and *Nr1d2*) than on RORE-containing genes such as *Bmal1*, *E4bp4*, and *Cry1* (Figure 5C), in line with the notion that the PERs can directly and indirectly affect *Bmal1* transcription [65], [66]. Overall, the network interactions in MMH-D3 cells appear to differ from those in the U2OS model in which *PER1* plays a more dominant role than *PER2* and *PER3* [27], and is expected to differ from those in 3T3 and 3T3-L1 models. Thus, extensive investigation into the network features of these cellular models will require additional experiments and is warranted in future studies, as we have done with the U2OS model [27].

### Concluding Remarks

Cell type-specific function of clock genes may result from their differential tissue expression and activity (i.e., expression levels, ratio of repressors to activators, rhythmicity, and relative amplitudes), compensatory mechanisms, alternative splice variants, and post-translational modifications (PMTs), all of which can be rendered cell type specific by local physiology. Recent studies have suggested a role for stoichiometric balance among clock proteins in circadian clock robustness and periodicity, and call for mechanistic studies in a tissue specific manner [67]–[69]. In the context of the liver and adipose tissue function, it is plausible that the basic core clock mechanism incorporates cell type-specific factors and forms distinctive functional networks to regulate (and in turn be regulated by) different local physiologies. It is interesting to note, as PTMs of clock factors (e.g., phosphorylation, ubiquitination, ADP-ribosylation, acetylation, and O-GlcNAcylation) represent critical regulatory mechanisms [33], [70], tissue-specific cellular functions and metabolic states that affect the PTMs would provide important inputs to adjust local circadian clocks, and *vice versa*. Thus, cell type specific clock gene function starts to make sense when local physiology is considered as inputs to the clock. This challenge surely provides an opportunity for deeper insights into mechanism of tissue specific clocks, akin to the recent realization that cyclin-dependent kinase networks in the cell cycle control program are tissue specific [71].

In summary, we established three new mouse cellular clock models: fibroblasts, adipocytes, and hepatocytes. These cellular clock models offer experimental tractability, efficiency, and versatility, which are more difficult or impossible to apply to traditional tissue or animal models. Of note, in contrast to previous cellular clock models, the new clock models are amenable to high throughput experiments with inexpensive off-the-shelf recording systems, making these lines especially suitable for screening small molecules or genomic entities for impacts on cell autonomous clocks relevant to metabolism. We validated these models by developing and testing an shRNA panel of selected known clock genes. Results from this study and others point to the previously under-appreciated cell type specificity of clock gene function in circadian physiology (Figure S6). The prevalence of tissue-specific clock gene function will have important implications for future studies of clock factors that affect local clock function. It is our hope that our findings in this study, along with the new cellular clock models, approaches, and tools developed here, can be applied to a greater variety of cell types in future studies, to reveal the full range of tissue-specific clock properties underlying local circadian biology.

## Materials and Methods

### Animals

*Per3^−/−^* mice were obtained from David Weaver at the University of Massachusetts. Knockout mice were bred with *mPer2^Luc^* reporter mice to obtain homozygous knockouts harboring the *mPer2^Luc^* reporter. Wheel-running assays were performed and analyzed as described previously [8]. All animal studies were conducted in accordance with the regulations of the Committees on Animal Care and Use at University of Memphis and UCSD.

### Cell and Tissue Culture

All cell culture media were from HyClone. 3T3 (also known as NIH 3T3) and 3T3-L1 cells were cultured in regular medium in which DMEM was supplemented with 10% FBS and 1× penicillin-streptomycin-glutamine (PSG). For 3T3-L1 differentiation, pre-adipocytes were first grown to confluence (Day 0). On Day 2, cells were fed with induction medium (regular medium with 1 µM dexamethasone, 0.5 mM isobutylmethylxanthine, and 2 µg/ml insulin). On Day 4, cells were changed to maintenance medium (regular medium containing 2 µg/ml insulin). From day 6 onward, cells were grown in regular medium until use. For bioluminescence recording, 3T3 and 3T3-L1 cells were grown in 25 mM HEPES-buffered regular medium (pH 7.4) containing 1 nM forskolin and 1 mM luciferin. Fully differentiated 3T3-L1 cells were used in all experiments.

MMH-D3 cells were grown in regular medium in which RPMI medium was supplemented with 10% FBS, 1× PSG, 10 µg/ml insulin, 55 ng/ml epidermal growth factor (EGF), and 16 ng/ml insulin like growth factor-II (IGF-II). For differentiation, pre-hepatocytes were first grown to 100% confluence. Two days later, cells were replaced with differentiation medium (regular medium with 2% DMSO). Medium change was repeated every 48 hours for 6–8 days for cells to be fully differentiated for use. Circadian rhythms of differentiated cells were synchronized with 200 nM dexamethasone followed by bioluminescence recording in 25 mM HEPES-buffered serum-free explant medium (pH 7.4) containing B-27 and 1 mM luciferin, as we have done previously [43]. Fully differentiated MMH-D3 cells were used in all experiments.

SCN explants and dissociated neuronal cells were prepared and cultured as previously described [43]. Bioluminescence recording of explants, single cell-imaging of individual SCN neurons, and respective data analysis were performed as previously described [43].

### Generation of Reporter Cell Lines

Lentiviral luciferase reporters of the *Per2* or *Bmal1* promoter were described previously [17], [43]. Reporter cells and clonal lines were generated as previously described [23]. Briefly, reporter viral particles of high titer (>10^8^ viral particles/ml) were obtained by ultracentrifugation and used to infect 3T3, 3T3-L1, and MMH-D3 cells. Clonal cell lines of homogenous cell populations were obtained by single cell sorting and cloning in 96 well plates. We then selected the clones that expressed high levels of luciferase and exhibited circadian properties comparable to infected parental cell populations. These brighter cells were used in high-throughput assays on 96 well plates. Knockdown phenotypes were confirmed to be independent of the reporter, either *Per2*-d*Luc* or *Bmal1*-d*Luc*, and thus phenotypic differences across cell types are unlikely due to different reporter insertion sites.

### Construction of Lentiviral shRNA Vectors and Viral Preparation

We used an optimized shRNA design algorithm adapted from Aza-Blanc et al. [72] for target sequence prediction. This adapted algorithm selects for optimal target sequence for knockdown, and against homologous sequences to minimize off-target effects. We selected 6 target sequences for each gene as listed in Table S5. Each shRNA construct contained a sense and an antisense target sequence of 19 nucleotides (nts) in length, separated by 9 nts for a hairpin loop, and flanked by TTTG at 5′ and GATC at 3′ ends for cohesive end cloning. All oligos (55 nts) were synthesized by Integrated DNA Technologies (IDT). The annealed oligonucleotides were first cloned into the BbsI and SpeI sites of a pGWL-si2/U6 vector, in which the shRNA expression cassette is driven by an RNA polymerase III- based mouse U6 promoter. Subsequently, the U6-shRNA cassette was cloned into the lentiviral pLL3.7GW vector (modified from pLL3.7) [17], [73] in a Gateway LR Clonase reaction (Life Technologies), according to manufacturer's instructions.

Viral particles were prepared using standard methods in 293T cells on 12-well plates as previously described [23], [74]. Culture medium containing viral particles (∼10^6^ viral particles/ml) were collected and used for subsequent infection of reporter cells. Transfection and infection efficiency were estimated by observing GFP co-expressed from a CMV promoter. To produce high titer viruses, crude viral particles were concentrated through ultracentrifugation and appropriate titers were used, as described previously [74]. This pipeline allowed us to generate a panel of shRNA constructs against all known clock factors for genetic perturbation and phenotyping.

### Bioluminescence Recording and Data Analysis

We used a LumiCycle luminometer (version 2.31, Actimetrics) for bioluminescence recording of cells grown on 35 mm culture dishes, as described elsewhere [17], [23], [43]. The LumiCycle Analysis program version 2.53 (Actimetrics) was used to determine circadian parameters. Briefly, raw data were fitted to a linear baseline, and the baseline-subtracted data were fitted to a sine wave (damped), from which period length and goodness of fit and damping constant were determined. For samples that showed persistent rhythms, goodness-of-fit of >80% was usually achieved. Due to high transient luminescence upon medium change, the first cycle was usually excluded from rhythm analysis. Damping rate = 1/damping constant. For amplitude analysis, raw data from day 3 to day 5 were fitted to a linear baseline, and the baseline-subtracted (polynomial number = 1) data were fitted to a sine wave, from which the amplitude was determined.

We used a Synergy 2 SL microplate reader (Bio Tek) for bioluminescence recording of cells grown on 96 well plates, as previously described [23]. Synergy data were analyzed with the MultiCycle Analysis program (Actimetrics), in which bioluminescence data were baseline-subtracted and fit to a damped sine wave to determine period length, goodness of fit, and amplitude, as with LumiCycle Analysis. Due to the various reporter expression levels and for direct comparison of different rhythms, baseline subtracted data were plotted. Because there is no damping rate output function in the MultiCycle Analysis, we used a curve fitting program of “CellulaRhythm” to determine damping rate from Synergy data as previously developed [75].

### Western Blot Analysis

For testing shRNA knockdown efficiency targeting each clock gene, the cDNA was cloned into a p3XFlag-CMV-14 vector. Flag-tagged cDNA was co-transfected with the indicated shRNA in 3T3 or 293T cells. Forty eight hours post-transfection, cells were lysed in RIPA buffer containing complete protease (Roche) and phosphatase inhibitors (Sigma). Protein expression was determined by Western blot analysis using an anti-Flag monoclonal antibody (Sigma). For all Western assays, PVDF membrane was used in protein transfer, and SuperSignal West Pico substrate (Thermo Scientific) was used for chemiluminescent detection.

### Quantitative PCR (qPCR) Analysis

For qPCR analysis, parallel infection experiments were performed as with bioluminescence recording. Cells were harvested prior to medium change and were therefore unsynchronized. Total RNAs were prepared using the RNeasy 96 kit (Qiagen), as previously described [31]. Reverse transcription was performed using a high-capacity RNA to cDNA kit (Applied Biosystems), and qPCR was performed using SYBR Green PCR master mix (Thermo Scientific) on an iCycler thermal cycler (BioRad). The primers used in qPCR analysis are listed in Table S6. Transcript levels for each gene were normalized to Gapdh and values were expressed as percentage of expression in NS control cells, as previously described [17].

## Supporting Information

Figure S1 Outline of generation of lentiviral shRNAs for gene knockdown.
(TIF)

Figure S2 Western blot analysis of shRNA-mediated gene knockdown.
(TIF)

Figure S3 Knockdowns of *Bmal2* and *Npas2* lead to no obvious circadian phenotypes. Bioluminescence expression patterns upon KD of *Bmal2* and *Npas2* in all three cell types. See Figure 2 for details.
(TIF)

Figure S4 Knockdowns of *Per1*, *Per2*, and *Per3* lead to cell type-specific circadian phenotypes. Bioluminescence expression patterns upon KD of *Per1* (A), *Per2* (B), and *Per3* (C) in 3T3 cells harboring the *Per2*-d*Luc* reporter and in 3T3-L1 and MMH-D3 cells harboring the *Bmal1*-d*Luc* reporter. KD of *Per1* leads to short period in MMH-D3 cells. KD of *Per2* leads to short period in 3T3 and MMH-D3 cells. KD of *Per3* leads to short period in all cell lines. These data are consistent with and complement those in Figure 4 where a different reporter was used for each cell type.
(TIF)

Figure S5 Bioluminescence expression patterns upon knockdowns of *Per1*, *Per2*, and *Per3* in MMH-D3 cell line. The raw data of *Per1*, *Per2*, and *Per3* KDs in MMH-D3 cells were plotted. The amplitude reduction upon knockdown is evident in both the raw data plots presented here and the subtracted data plots in Figure 4.
(TIF)

Figure S6 Summary of knockdown and knockout circadian phenotypes. Phenotypes of 3T3, 3T3-L1, MMH-D3, and *Per3^−/−^* SCN neurons are from this study, and those of other cells and tissues are from several previous studies [27], [31], [43], [45], [46], [50], [54], [58]. Loss-of-function of *Bmal1* and *Clock* leads to arrhythmic phenotype, but this clock defect may be masked in the SCN. *Cry1* is also required in cell-autonomous preparations, but its KD can lead to arrhythmicity, rapid damping or short period, depending on KD efficiency. *Cry2* loss-of-function, on the other hand, leads to long period phenotypes in all cells and tissues examined. In comparison, the *Per* genes show tissue- or cell type-specific phenotypes. MEF, mouse embryonic fibroblasts; MAF, mouse adult fibroblasts; neuron, dissociated SCN neurons. WT, wild type; long, longer period than WT; short, shorter period than WT; LA, rhythmic but low amplitude; RD, transiently rhythmic and rapid damping; AR, arrhythmic.
(TIF)

Table S1 Parameter analysis of knockdowns in 3T3 cells.
(DOCX)

Table S2 Parameter analysis of knockdowns in 3T3-L1 cells.
(DOCX)

Table S3 Parameter analysis of knockdowns in MMH-D3 cells.
(DOCX)

Table S4 Parameter analysis of *Per* composite knockdowns in MMH-D3 cells.
(DOCX)

Table S5 List of target sequences against a panel of known clock genes.
(DOCX)

Table S6 List of primers used in qPCR analysis.
(DOCX)
