# Supplementary figures and images for: Cell Type-Specific Functions of Period Genes Revealed by Novel Adipocyte and Hepatocyte Circadian Clock Models
Source: PLoS Genet. 2014 Apr 3;10(4):e1004244. doi: 10.1371/journal.pgen.1004244 (PMC3974647; doi:10.1371/journal.pgen.1004244)

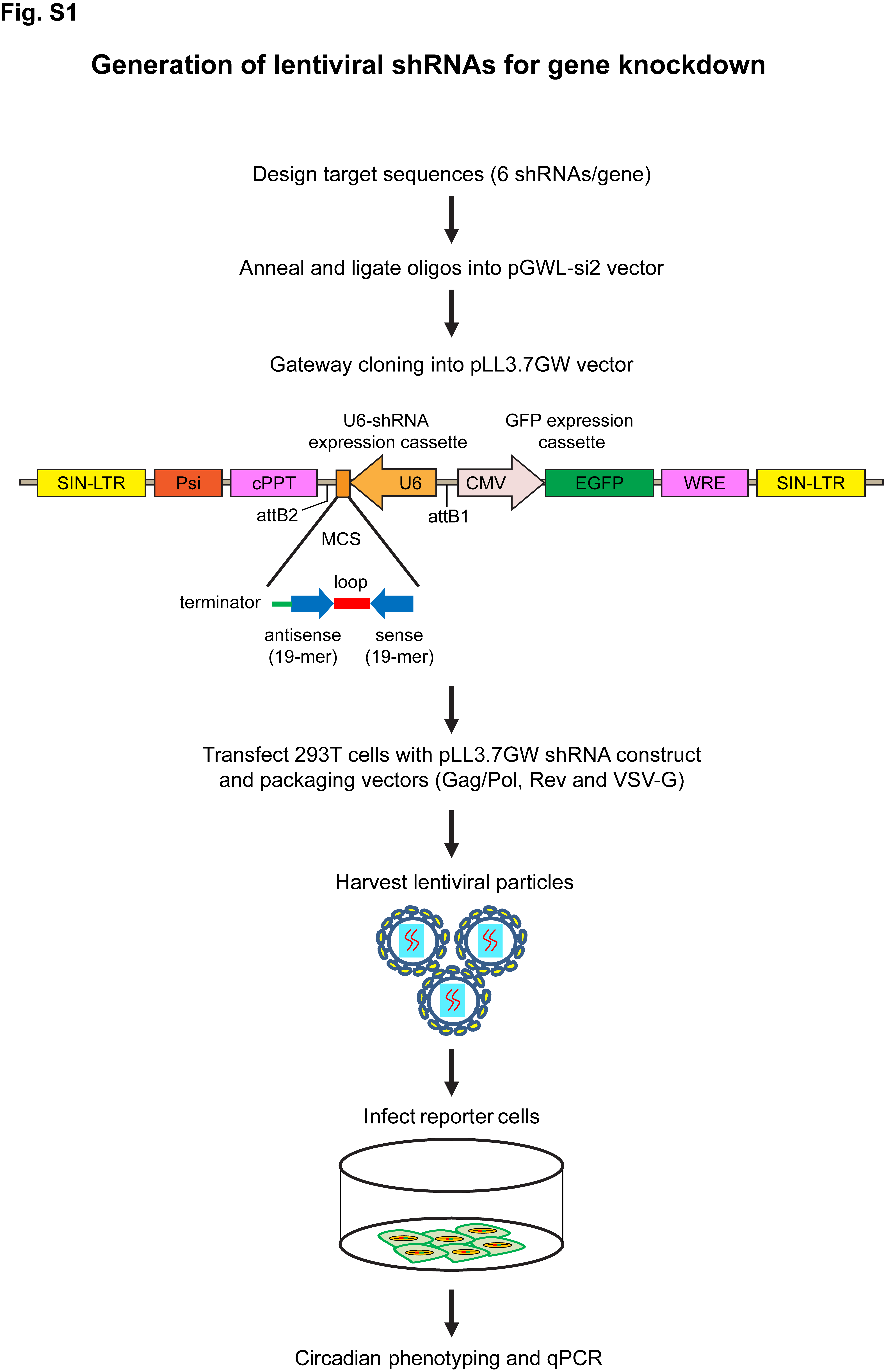

Supplement: Figure S1 — Outline of generation of lentiviral shRNAs for gene knockdown. (TIF) [file pgen.1004244.s001.tif]

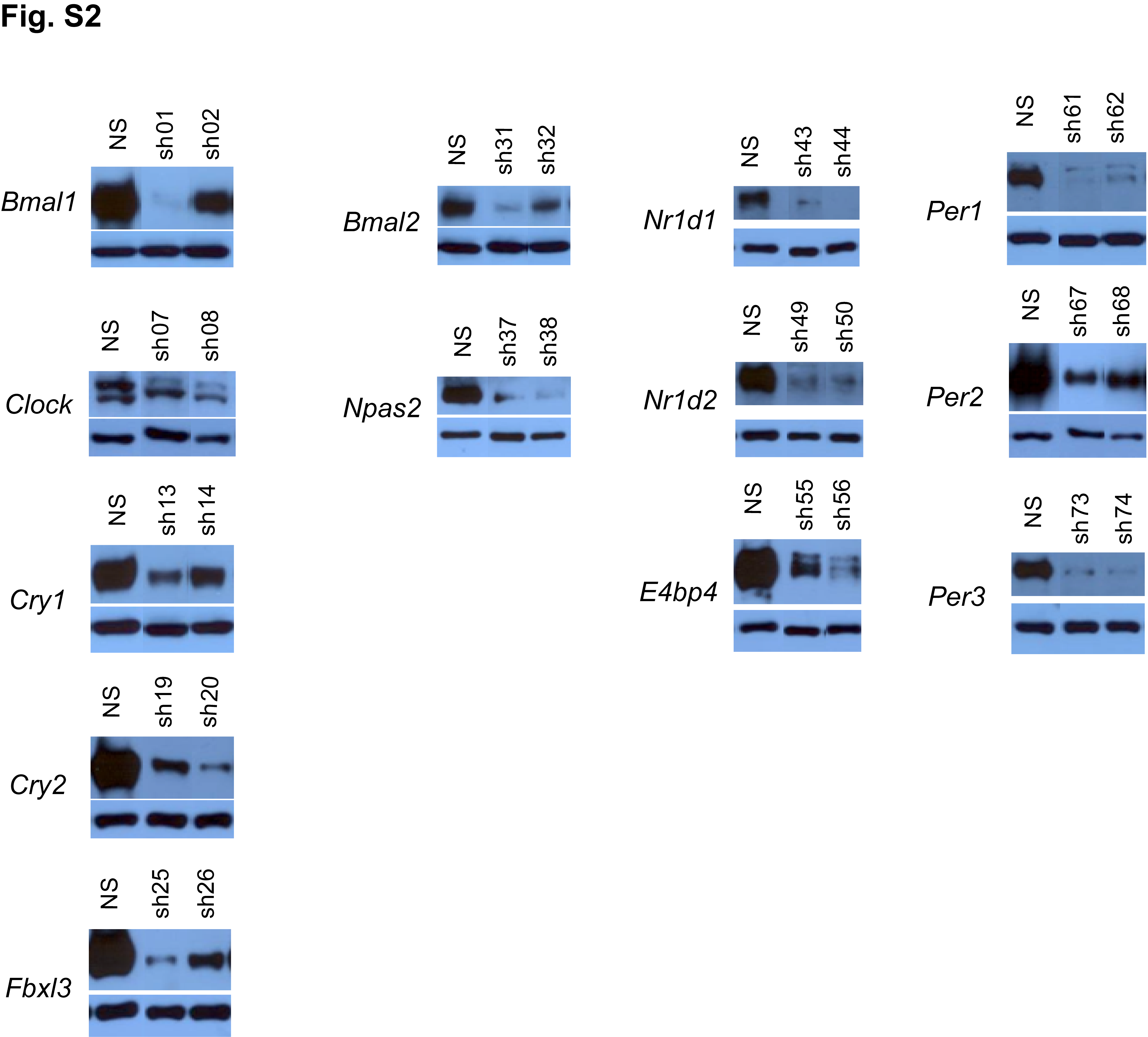

Supplement: Figure S2 — Western blot analysis of shRNA-mediated gene knockdown. (TIF) [file pgen.1004244.s002.tif]

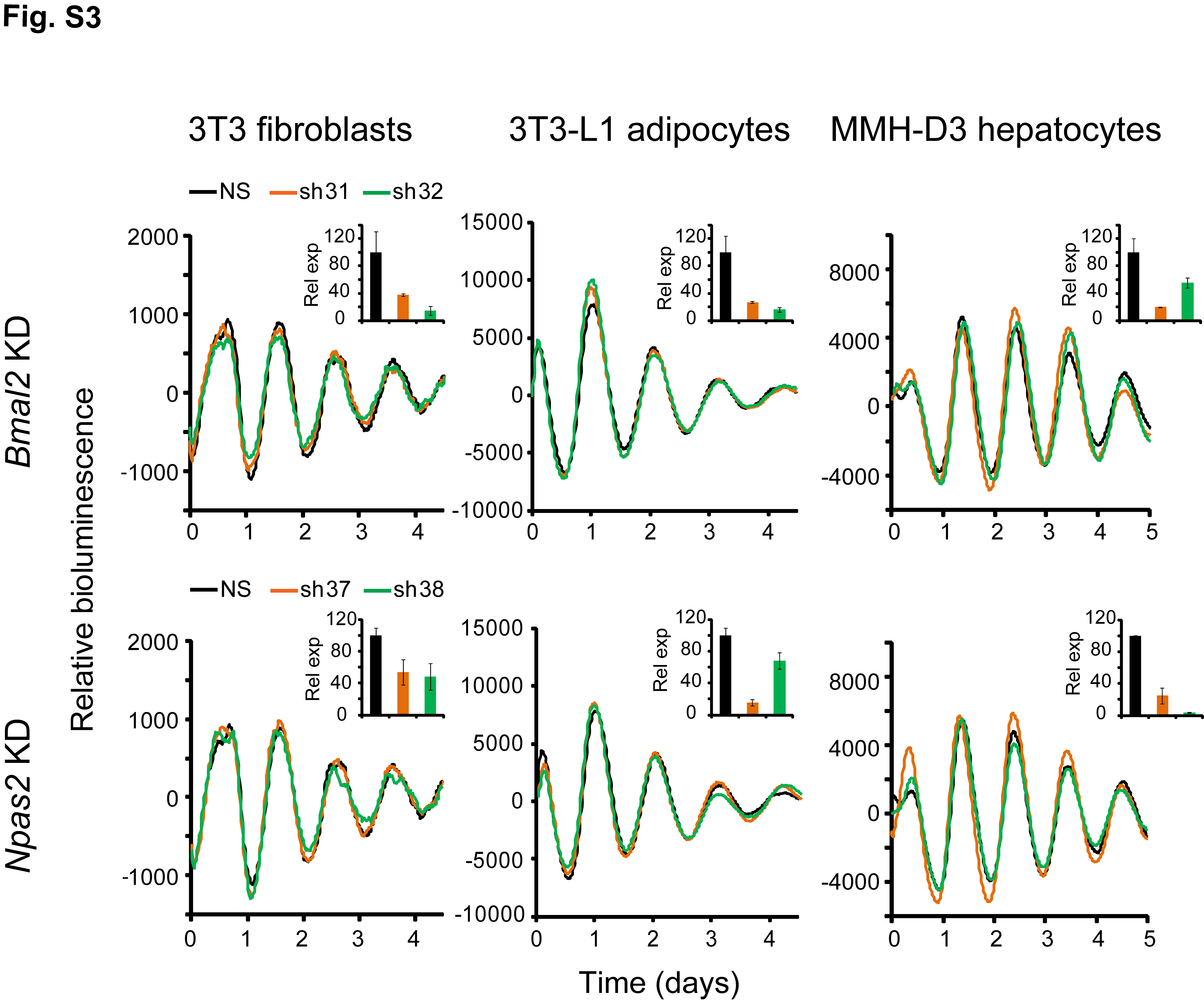

Supplement: Figure S3 — Knockdowns of Bmal2 and Npas2 lead to no obvious circadian phenotypes. Bioluminescence expression patterns upon KD of Bmal2 and Npas2 in all three cell types. See Figure 2 for details. (TIF) [file pgen.1004244.s003.tif]

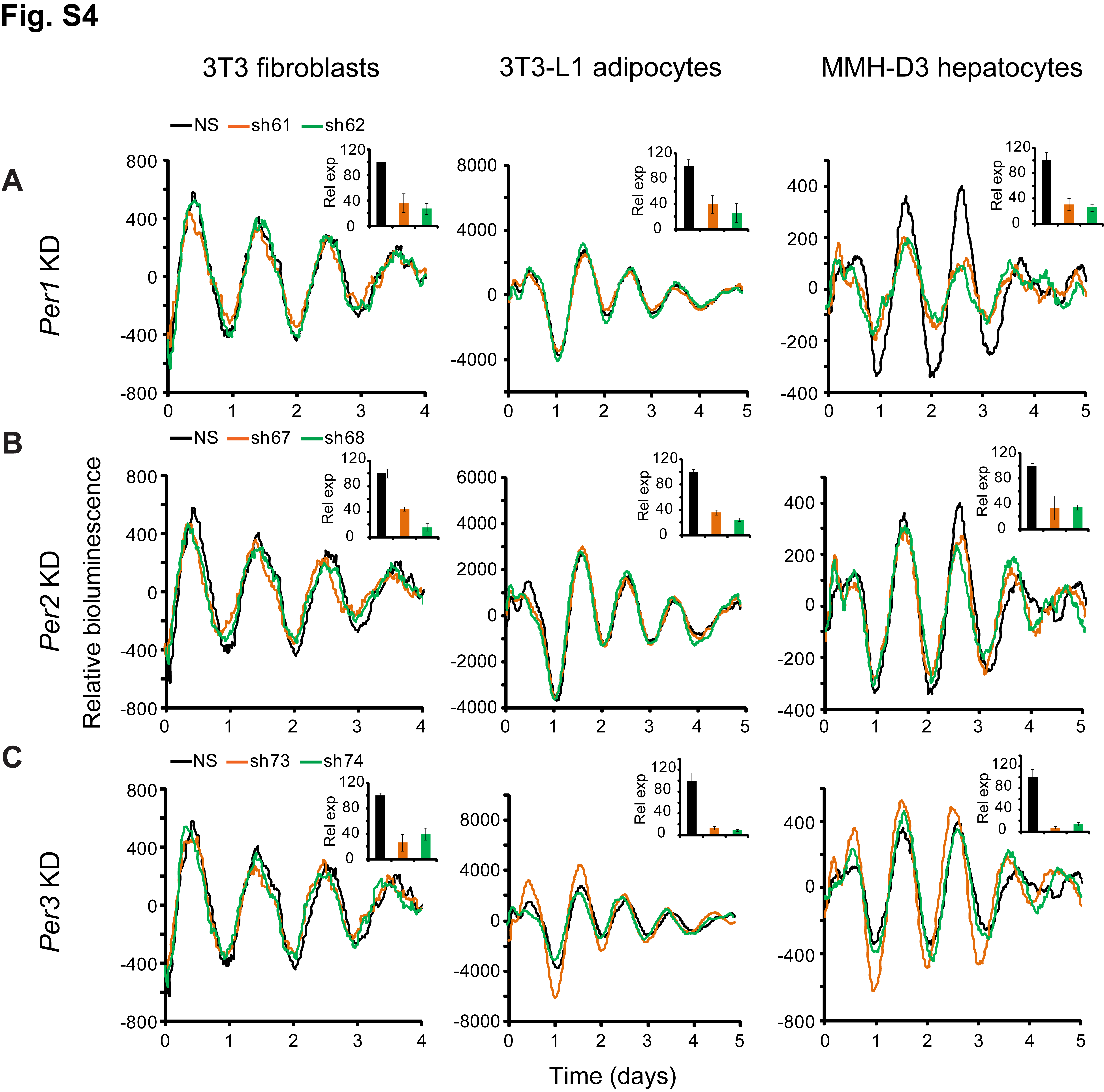

Supplement: Figure S4 — Knockdowns of Per1, Per2, and Per3 lead to cell type-specific circadian phenotypes. Bioluminescence expression patterns upon KD of Per1 (A), Per2 (B), and Per3 (C) in 3T3 cells harboring the Per2-dLuc reporter and in 3T3-L1 and MMH-D3 cells harboring the Bmal1-dLuc reporter. KD of Per1 leads to short period in MMH-D3 cells. KD of Per2 leads to short period in 3T3 and MMH-D3 cells. KD of Per3 leads to short period in all cell lines. These data are consistent with and complement those in Figure 4 where a different reporter was used for each cell type. (TIF) [file pgen.1004244.s004.tif]

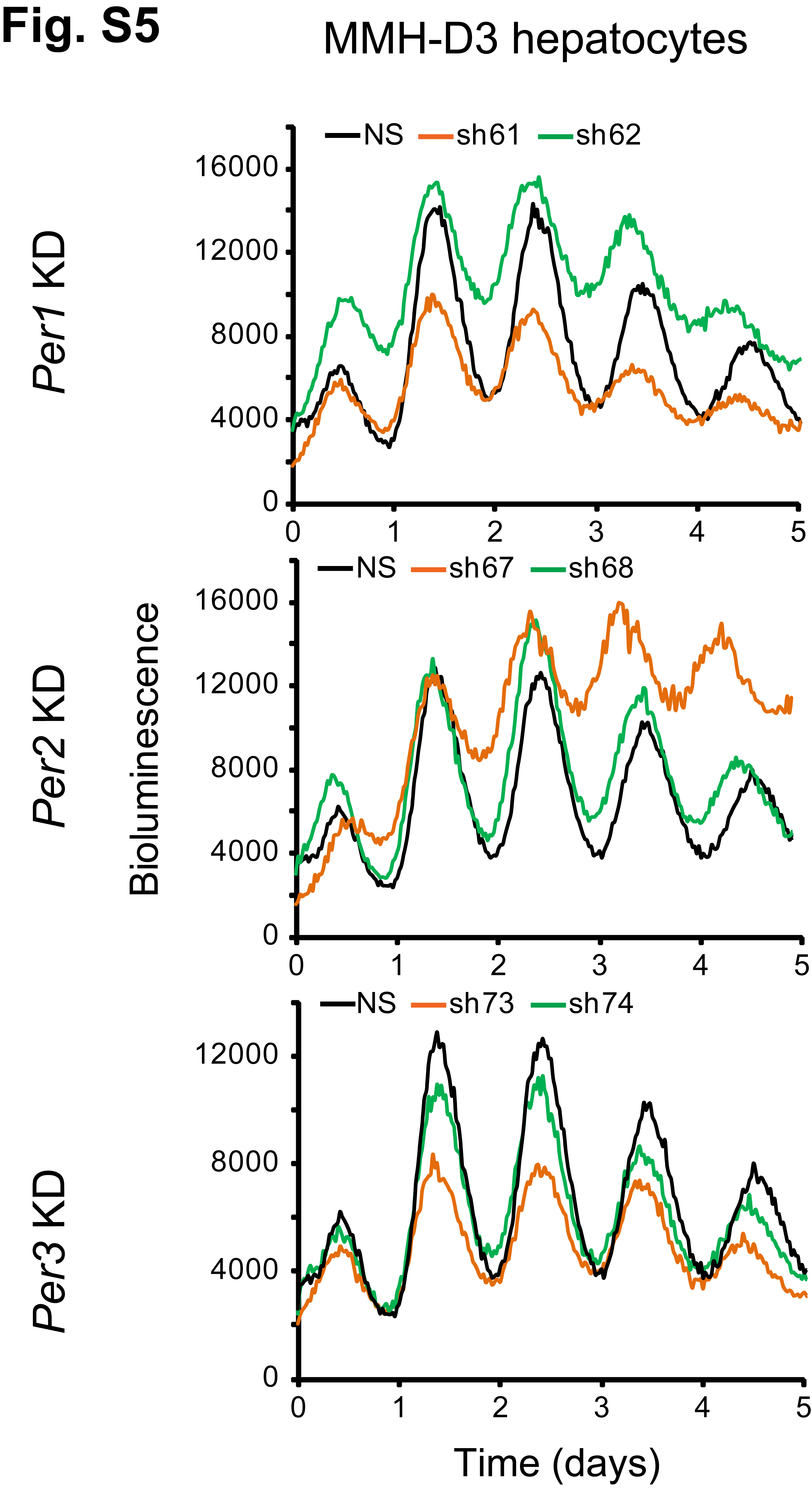

Supplement: Figure S5 — Bioluminescence expression patterns upon knockdowns of Per1, Per2, and Per3 in MMH-D3 cell line. The raw data of Per1, Per2, and Per3 KDs in MMH-D3 cells were plotted. The amplitude reduction upon knockdown is evident in both the raw data plots presented here and the subtracted data plots in Figure 4. (TIF) [file pgen.1004244.s005.tif]

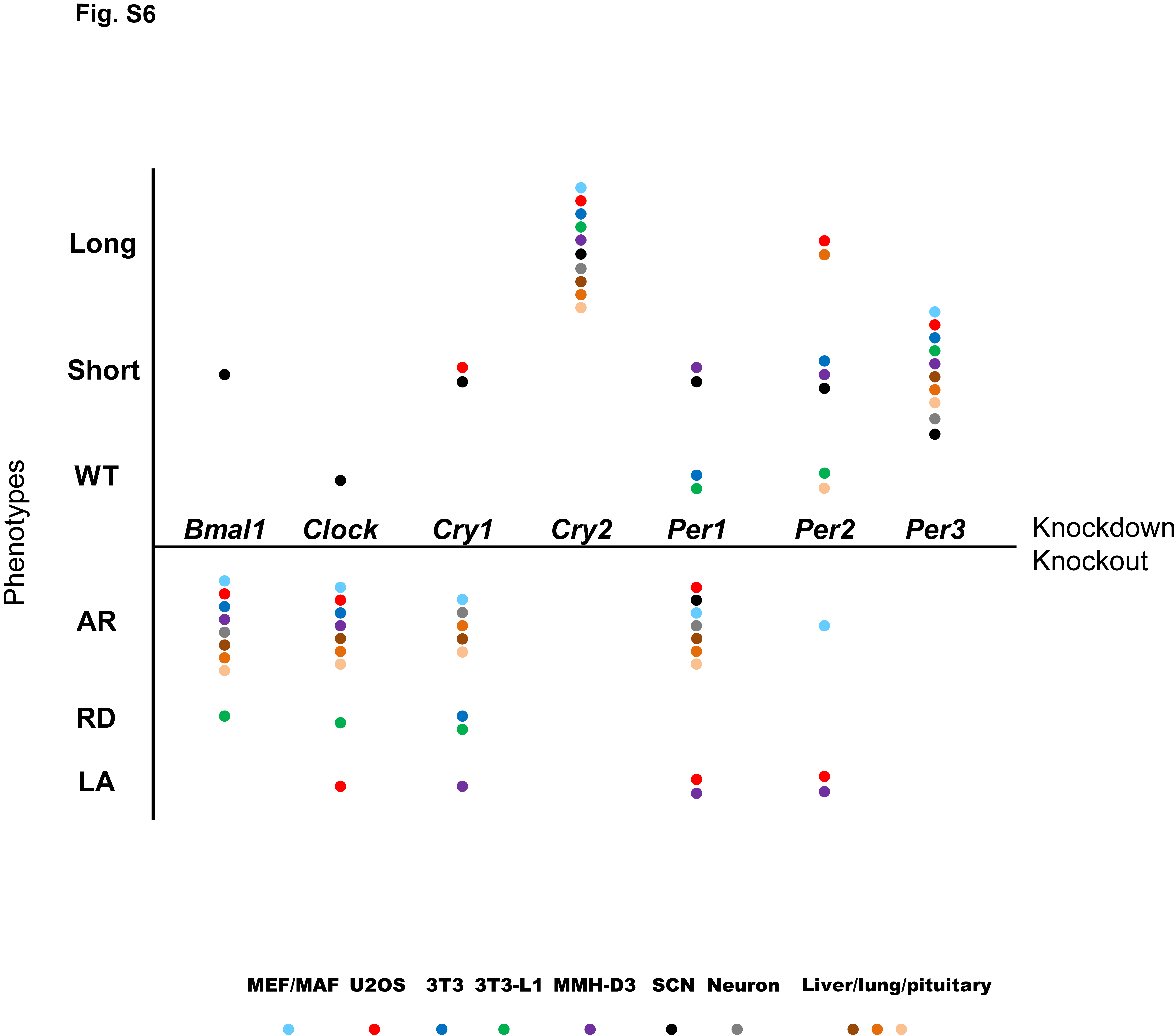

Supplement: Figure S6 — Summary of knockdown and knockout circadian phenotypes. Phenotypes of 3T3, 3T3-L1, MMH-D3, and Per3−/− SCN neurons are from this study, and those of other cells and tissues are from several previous studies [27], [31], [43], [45], [46], [50], [54], [58]. Loss-of-function of Bmal1 and Clock leads to arrhythmic phenotype, but this clock defect may be masked in the SCN. Cry1 is also required in cell-autonomous preparations, but its KD can lead to arrhythmicity, rapid damping or short period, depending on KD efficiency. Cry2 loss-of-function, on the other hand, leads to long period phenotypes in all cells and tissues examined. In comparison, the Per genes show tissue- or cell type-specific phenotypes. MEF, mouse embryonic fibroblasts; MAF, mouse adult fibroblasts; neuron, dissociated SCN neurons. WT, wild type; long, longer period than WT; short, shorter period than WT; LA, rhythmic but low amplitude; RD, transiently rhythmic and rapid damping; AR, arrhythmic. (TIF) [file pgen.1004244.s006.tif]
