# Supplementary material for: Cell Type-Specific Functions of Period Genes Revealed by Novel Adipocyte and Hepatocyte Circadian Clock Models
Source: PLoS Genet. 2014 Apr 3;10(4):e1004244. doi: 10.1371/journal.pgen.1004244 (PMC3974647; doi:10.1371/journal.pgen.1004244)
Supplement: Table S1 — Parameter analysis of knockdowns in 3T3 cells. (DOCX) [file pgen.1004244.s007.docx]

**Table S1. Parameter analysis of knockdowns in 3T3 cells.**

| Gene KD | Period (hr) | Amplitude | Goodness of  fit (%) | Damping rate | Phenotype |
| --- | --- | --- | --- | --- | --- |
| NS | 24.22 ± 0.27 | 463.77 ± 176.27 | 93.79 ± 1.35 | 0.02 ± 0.00 | WT |
| *Bmal1* | 40.05 ± 9.48 | 144.75 ± 95.79 | 41.85 ± 21.84 | 0.03 ± 0.03 | AR |
| *Bmal2* | 24.36 ± 0.31 | 408.95 ± 193.19 | 94.02 ± 1.50 | 0.02 ± 0.00 | WT |
| *Clock* | 32.75 ± 13.23 | 122.33 ± 89.53 | 39.74 ± 38.96 | 0.03 ± 0.04 | AR |
| *Npas2* | 24.46 ± 0.18 | 466.41 ± 235.47 | 94.66 ± 0.76 | 0.02 ± 0.00 | WT |
| *Cry1* | 24.14 ± 0.63 | 247.32 ± 133.81 | 79.39 ± 20.19 | 0.03 ± 0.01* | RD |
| *Cry2* | 25.58 ± 0.25** | 615.71 ± 199.87 | 94.61 ± 1.01 | 0.01 ± 0.00 | Long |
| *Per1* | 24.52 ± 0.52 | 332.71 ± 138.56 | 93.30 ± 0.73 | 0.02 ± 0.01 | WT |
| *Per2* | 23.53 ± 0.29* | 262.18 ± 102.56 | 92.75 ± 1.81 | 0.02 ± 0.00 | Short |
| *Per3* | 22.70 ± 0.39** | 347.94 ± 168.41 | 91.47 ± 2.20 | 0.02 ± 0.00 | Short |
| *Fbxl3* | 25.43 ± 0.91* | 189.14 ± 112.96* | 91.45 ± 2.01 | 0.02 ± 0.01 | Long, LA |
| *Nr1d1* | 23.99 ± 0.63 | 362.59 ± 211.63 | 83.96 ± 13.34 | 0.02 ± 0.00 | WT |
| *Nr1d2* | 24.36 ± 0.60 | 258.66 ± 140.72* | 92.72 ± 3.81 | 0.02 ± 0.01 | LA |
| *E4bp4* | 23.16 ± 0.57* | 112.67 ± 152.85 | 72.64 ± 15.05 | 0.05 ± 0.00** | Short, RD |

Notes:

MultiCycle Analysis and CellulaRhythm programs were used for data analysis (see Materials and Methods for detail). Circadian parameters shown are from one of the six shRNAs that gave the best KD efficiency and phenotypes. Mean ± SD are from four independent Synergy assay experiments on 96 well plates. *p < 0.05, **p < 0.0001 compared to NS control, *t*-test.
