# Supplementary material for: Cell Type-Specific Functions of Period Genes Revealed by Novel Adipocyte and Hepatocyte Circadian Clock Models
Source: PLoS Genet. 2014 Apr 3;10(4):e1004244. doi: 10.1371/journal.pgen.1004244 (PMC3974647; doi:10.1371/journal.pgen.1004244)
Supplement: Table S2 — Parameter analysis of knockdowns in 3T3-L1 cells. (DOCX) [file pgen.1004244.s008.docx]

**Table S2. Parameter analysis of knockdowns in 3T3-L1 cells.**

| Gene  KD | Period (hr) | Amplitude | Goodness of  fit (%) | Damping  rate | Phenotype |
| --- | --- | --- | --- | --- | --- |
| NS | 25.32 ± 0.27 | 1139.59 ± 48.95 | 92.30 ± 1.82 | 0.03 ± 0.00 | WT |
| *Bmal1* | 26.38 ± 0.46 | 388.21 ± 77.93 | 78.33 ± 5.35 | 0.05 ± 0.00* | RD |
| *Bmal2* | 25.48 ± 0.18 | 1043.62 ± 88.84 | 84.11 ± 2.25 | 0.03 ± 0.00 | WT |
| *Clock* | 31.13 ± 8.84 | 145.02 ± 37.03 | 73.82 ± 4.86 | 0.04 ± 0.01* | RD |
| *Npas2* | 25.38 ± 0.35 | 1011.03 ± 46.96 | 86.99 ± 7.22 | 0.03 ± 0.00 | WT |
| *Cry1* | 25.98 ± 0.35 | 548.80 ± 64.79 | 76.60 ± 2.94 | 0.04 ± 0.01* | RD |
| *Cry2* | 26.08 ± 0.35* | 901.21 ± 196.81 | 85.42 ± 8.46 | 0.03 ± 0.00 | Long |
| *Per1* | 25.78 ± 0.35 | 885.64 ± 129.13 | 92.39 ± 0.61 | 0.03 ± 0.00 | WT |
| *Per2* | 25.27 ± 0.00 | 810.29 ± 14.77 | 90.48 ± 1.08 | 0.03 ± 0.00 | WT |
| *Per3* | 24.57 ± 0.18* | 958.51 ± 105.09 | 88.32 ± 0.15 | 0.03 ± 0.00 | Short |
| *Fbxl3* | 25.98 ± 0.17* | 1173.21 ± 76.68 | 90.37 ± 2.53 | 0.03 ± 0.00 | Long |
| *Nr1d1* | 25.38 ± 0.35 | 1235.21 ± 68.56 | 86.99 ± 7.22 | 0.03 ± 0.00 | WT |
| *Nr1d2* | 24.67 ± 0.00* | 1715.94 ± 209.78 | 89.83 ± 1.20 | 0.02 ± 0.00 | Short |
| *E4bp4* | 25.27 ± 0.31 | 576.54 ± 151.48* | 86.65 ± 2.93 | 0.03 ± 0.00 | LA |

Notes:

MultiCycle Analysis and CellulaRhythm programs were used for data analysis (see Materials and Methods for detail). Circadian parameters shown are from one of the six shRNAs that gave the best KD efficiency and phenotypes. Plate-to-plate variation is less tight in 3T3-L1 cells than in 3T3 and MMH-D3 cells, and mean ± SD shown here for 3T3-L1 represent three samples/wells from one of four Synergy assay experiments on 96 well plates. *p < 0.001 compared to NS control, *t*-test.
