# Supplementary material for: Cell Type-Specific Functions of Period Genes Revealed by Novel Adipocyte and Hepatocyte Circadian Clock Models
Source: PLoS Genet. 2014 Apr 3;10(4):e1004244. doi: 10.1371/journal.pgen.1004244 (PMC3974647; doi:10.1371/journal.pgen.1004244)
Supplement: Table S3 — Parameter analysis of knockdowns in MMH-D3 cells. (DOCX) [file pgen.1004244.s009.docx]

**Table S3. Parameter analysis of knockdowns in MMH-D3 cells.**

| Gene KD | Period (hr) | Amplitude | Goodness of  fit (%) | Damping  rate | Phenotype |
| --- | --- | --- | --- | --- | --- |
| NS | 24.71 ± 1.03 | 1887.01 ± 371.47 | 92.28 ± 0.56 | 0.00 ± 0.00 | WT |
| *Bmal1* | 43.75 ± 7.35 | 520.23 ± 206.57 | 27.05 ± 27.90 | 0.08 ± 0.02 | AR |
| *Bmal2* | 24.86 ± 0.69 | 1933.81 ± 184.92 | 90.28 ± 1.35 | 0.00 ± 0.01 | WT |
| *Clock* | 25.17 ± 2.017 | 159.41 ± 75.79 | 3.86 ± 46.72 | 0.03 ± 0.03 | AR |
| *Npas2* | 24.87 ± 0.97 | 1815.42 ± 279.01 | 92.53 ± 0.16 | 0.00 ± 0.01 | WT |
| *Cry1* | 24.36 ± 0.00 | 767.50 ± 104.14* | 83.20 ± 14.33 | 0.01 ± 0.02 | LA |
| *Cry2* | 26.99 ± 0.70* | 2986.70 ± 640.86* | 93.77 ± 0.07 | 0.00 ± 0.00 | Long, HA |
| *Per1* | 23.04 ± 0.43* | 423.38 ± 314.22* | 87.91 ± 4.15 | 0.00 ± 0.01 | Short, LA |
| *Per2* | 23.22 ± 0.33* | 681.88 ± 447.39* | 80.51 ± 23.11 | 0.00 ± 0.02 | Short, LA |
| *Per3* | 23.25 ± 0.13* | 1110.79 ± 477.37 | 93.41 ± 0.64 | 0.00 ± 0.01 | Short |
| *Fbxl3* | 27.70 ± 2.89 | 229.83 ± 49.12* | 83.13 ± 2.98 | 0.02 ± 0.00* | RD, LA |
| *Nr1d1* | 24.77 ± 1.15 | 1068.77 ± 273.51 | 94.47 ± 1.31 | 0.00 ± 0.00 | WT |
| *Nr1d2* | 23.28 ± 0.13* | 1321.39 ± 354.14 | 91.78 ± 3.02 | 0.00 ± 0.01 | Short |
| *E4bp4* | 23.17 ± 0.15* | 1238.96 ± 416.09 | 92.84 ± 2.95 | 0.00 ± 0.00 | Short |

Notes:

MultiCycle Analysis and CellulaRhythm programs were used for data analysis (see Materials and Methods for detail). Circadian parameters shown are from one of the six shRNAs that gave the best KD efficiency and phenotypes. Mean ± SD are from four independent Synergy assay experiments on 96 well plates. *p < 0.05 compared to NS control, *t*-test.
