# Supplementary material for: Cell Type-Specific Functions of Period Genes Revealed by Novel Adipocyte and Hepatocyte Circadian Clock Models
Source: PLoS Genet. 2014 Apr 3;10(4):e1004244. doi: 10.1371/journal.pgen.1004244 (PMC3974647; doi:10.1371/journal.pgen.1004244)
Supplement: Table S4 — Parameter analysis of Per composite knockdowns in MMH-D3 cells. (DOCX) [file pgen.1004244.s010.docx]

**Table S4. Parameter analysis of *Per* composite knockdowns in MMH-D3 cells.**

| Gene  KD | Period (hr) | Amplitude | Goodness of  fit (%) | Damping  rate | Phenotype |
| --- | --- | --- | --- | --- | --- |
| NS | 25.60 ± 0.62 | 1185.41 ± 1.40 | 90.85 ± 0.53 | 0.37 ± 0.04 | WT |
| *Per1* | 23.30 ± 0.00* | 806.97 ± 0.10 | 91.24 ± 0.43 | 0.51 ± 0.03 | Short |
| *Per2* | 23.23 ± 0.06* | 357.32 ± 0.46 | 80.50 ± 1.90 | 0.75 ± 0.06* | Short, RD |
| *Per3* | 23.53 ± 0.06* | 1150.24 ± 0.20 | 91.56 ± 0.79 | 0.43 ± 0.02 | Short |
| *Per1/Per2* | ND | 228.62 ± 10.16 | 61.01 ± 0.00 | ND | AR |
| *Per1/Per3* | 23.13 ± 0.42* | 854.99 ± 0.25 | 88.20 ± 5.59 | 0.43 ± 0.05 | Short |
| *Per2/Per3* | 23.23 ± 0.23* | 336.56 ± 0.23 | 85.83 ± 4.80 | 0.68 ± 0.04* | Short, RD |
| *Per1/2/3* | ND | 287.43 ± 16.05 | 61.01 ± 0.00 | ND | AR |

Notes:

LumiCycle Analysis program was used for data analysis (see Materials and Methods for detail). Mean ± SD are from three samples/dishes for each KD in a LumiCycle assay. sh62, sh67 and sh74 were used to knock down *Per1*, *Per2* and *Per3*, respectively. *p < 0.001 compared to NS control, *t*-test. ND, not determined due to arrhythmicity.
