# Supplementary material for: Cell Type-Specific Functions of Period Genes Revealed by Novel Adipocyte and Hepatocyte Circadian Clock Models
Source: PLoS Genet. 2014 Apr 3;10(4):e1004244. doi: 10.1371/journal.pgen.1004244 (PMC3974647; doi:10.1371/journal.pgen.1004244)
Supplement: Table S5 — List of target sequences against a panel of known clock genes. (DOCX) [file pgen.1004244.s011.docx]

Table S5. List of genes and six shRNA target sequences.

| shRNA ID | Genes | Target sequences | Accession # |
| --- | --- | --- | --- |
| NS |  | GCAACAAGATGAAGAGCAC |  |
| 01 | *Bmal1* | GCATCGATATGATAGATAA | NM_007489 |
| 02 |  | GTCGATGGTTCAGTTTCAT |  |
| 03 |  | GCACATGGTTCCACAACCA |  |
| 04 |  | Gcctcatggaaggttagaa |  |
| 05  06 |  | Ggaaggatcaagaatgcaa  ggatggctgttcagcacat |  |
| 07 | *Clock* | GGTCGTCCTTCAGCAGTCA | NM_007715 |
| 08 |  | GTTTACATCTAGACACAGT |  |
| 09 |  | GTCAGTCCATAAACTCCCA |  |
| 10 |  | GCTTCCTGGTAACGCGAGA |  |
| 11  12 |  | GGATGATAGAGGCAAATAT  GAGAACATTCAGAGGTTTA |  |
| 13 | *Cry1* | gccaagtgtttgataggag | NM_007771 |
| 14 |  | GTCAGTCCATAAACTCCCA |  |
| 15 |  | GCGGTTGCCTGTTTCCTGA |  |
| 16 |  | GGTGACCTGTGGATCAGCT |  |
| 17  18 |  | GGTCCCTTCTAACTCTAAT  GGGAGAAGCTCCGCGGGCA |  |
| 19 | *Cry2* | gaattcgcgtctgtttgta | NM_001113333 |
| 20 |  | GGTTCCTACTGCAATCTCT |  |
| 21 |  | GGAACCTCCTGGTGAAGAA |  |
| 22 |  | GCCAGCTGATGTGTTCCCA |  |
| 23  24 |  | GTTTGACCTTTGAATATGA  ggatgaatgccaattcctt |  |
| 25 | *Fbxl3* | GGACAGCAGCAAAGAATCA | NM_015822 |
| 26 |  | GCCTCTTGATGAAGAGTTA |  |
| 27 |  | GAATTGGAATCAGGTATTT |  |
| 28 |  | GCTTGTGAATTGCTCTTTA |  |
| 29  30 |  | GCATGTTCGCTTAGAACAT  gctttatggatctaccaaA |  |
| 31 | *Bmal2* | GCTGGCCTCTGAATGTTGT | NM_172309 |
| 32 |  | GCGGTGCAGTACTTGAGGT |  |
| 33 |  | GAAGACTCATTTAGACAAT |  |
| 34 |  | GCATCAATGTGCCCGGTGT |  |
| 35  36 |  | GGTTCTGAGTTTACAGAGA  GCTCCAGACGATCTTTCTT |  |
| 37 | *Npas2* | GTCATCGGATTCTTGCAGA | NM_008719 |
| 38 |  | GCTTGAACCCAAAGGAATT |  |
| 39 |  | GCCCTGACTTCGGCCATGA |  |
| 40 |  | GGAATTCACTTCGAGGCAT |  |
| 41  42 |  | GAACCCACCTCCACTCCAA  GCCGTTCACTGAGCAGCCT |  |
| 43 | *Nr1d1* | gcaaggcaacaccaagaat | NM_145434 |
| 44 |  | GCATCGTTGTTCAACGTGA |  |
| 45 |  | GACCTCACCTACTCCACAT |  |
| 46 |  | GGCTCAGCGTCATAATGAA |  |
| 47  48 |  | GTCCCTGACTCAAGGTTGT  GAAGACTTCTCTATGAGCT |  |
| 49 | *Nr1d2* | GGAGGAACATAATGCATTA | NM_011584 |
| 50 |  | Gcaatgaagaccatgatga |  |
| 51 |  | GTAGGTGGATGTTCTCAGA |  |
| 52 |  | GTTCTACTGTGTAAAGTCT |  |
| 53  54 |  | GTTCCATCATGAGGATGAA  GAGAACGGATTCCCAGGAA |  |
| 55 | *E4bp4* | gcaggtgacgaacattcaa | NM_017373 |
| 56 |  | GAAGTTGCATCTCAGTCAT |  |
| 57 |  | GGTGAAGATTTGCTCCTGA |  |
| 58 |  | GCTGAGAATTTGTATTTGA |  |
| 59  60 |  | GGGAATTCATTCCGGACGA  GAGCTGCTCTCCCTGAAAT |  |
| 61 | *Per1* | GCTGAAGTGGTCTGTCCAA | NM_011065 |
| 62 |  | GATGCAGCCTCTGTGCTGA |  |
| 63 |  | GACCAAGCCTCGTTAGCCT |  |
| 64 |  | GCTCCACCATCGTCCATGT |  |
| 65  66 |  | GACCAAGATTCGGGTCTCA  GGTGCTCCCTAACTATCTA |  |
| 67 | *Per2* | GCAGGAAGATATCTTTCAT | NM_011066 |
| 68 |  | GGCATTACCTCCGAGTATA |  |
| 69 |  | GGCCCTTGATGCTCGCCAT |  |
| 70 |  | GGCTGTGTTTACTGCGAGA |  |
| 71  72 |  | GAGATTCGCTACCAGCCCT  gcacacaaagaactgataa |  |
| 73 | *Per3* | GTGTTGCCGTGGCGTGTAA | NM_011067 |
| 74 |  | GTTCACCGTTACAGTTAAA |  |
| 75 |  | GCCGGAAGGTCTCCTTCAT |  |
| 76 |  | GCCGGTTCATGCTAGTGCT |  |
| 77  78 |  | GCGTGCACTTTGTCGACCT  GCTCTGGCTTCTGAACATA |  |
