# Supplementary material for: Cell Type-Specific Functions of Period Genes Revealed by Novel Adipocyte and Hepatocyte Circadian Clock Models
Source: PLoS Genet. 2014 Apr 3;10(4):e1004244. doi: 10.1371/journal.pgen.1004244 (PMC3974647; doi:10.1371/journal.pgen.1004244)
Supplement: Table S6 — List of primers used in qPCR analysis. (DOCX) [file pgen.1004244.s012.docx]

| Genes | Forward primer | Reverse primer |
| --- | --- | --- |
| *Bmal1* | ACCAACCCATACACAGAAGC | GACAGACTCGGAGACAAAGAG |
| *Bmal2* | CAGAGTGAAGGATGGTGCC | CCGCAATGAAGCAAAGACAG |
| *Clock* | ATGGTGTTTACCGTAAGCTGTAG | CTCGCGTTACCAGGAAGCAT |
| *Npas2* | ATGTTCGAGTGGAAAGGAGAC | CAAGTGCATTAAAGGGCTGTG |
| *Cry1* | ACTGAGGCACTTACACGTTTGG | GCAGGGAGTTTGCATTCATTC |
| *Cry2* | GGTTCCTACTGCAATCTCTGG | GTCATATTCAAAGGTCAAACGGG |
| *Fbxl3* | CCATCCCTTAAAGTCCTCGTG | TTCTCGTAAGCCATGACACTG |
| *Nr1d1* | GACCTTTCTCAGCACGACC | CATCACTGTCTGGTCCTTCAC |
| *Nr1d2* | CCCAAGAACGCTGATATCTCTAG | ACACAGTAGAACCATGCCAC |
| *E4bp4* | CTCTTTCTCCACTTACTCCCAC | TTGTTCGTCTTCCCCATCAG |
| *Per1* | TGTGTCAAGCAGGTTCAGG | TGTCCTGGTTTCGAAGTGTG |
| *Per2* | TGTTCCGACATGCTTGCG | GAAACAGCTTCCTCTGCTCCAG |
| *Per3* | CCCTACGGTTGCTATCTTCAG | CTTTCGTTTGTGCTTCTGCC |
| *Gapdh* | GCCTTCCGTGTTCCTACC | CCTCAGTGTAGCCCAAGATG |

**Table S6. List of primers used in qPCR.**
